# Mapping the Evolution of IBD Treatment: A Bibliometric Study on Biologics and Small Molecules

**DOI:** 10.3390/ph18030312

**Published:** 2025-02-24

**Authors:** Huibo Li, Jia Wang, Yang Hu, Wei Hu, Jun Li, Yang Liu, Rongsheng Zhao, Yi Zhun Zhu

**Affiliations:** 1 School of Pharmacy, Faculty of Medicine & Laboratory of Drug Discovery from Natural Resources and Industrialization, Macau University of Science and Technology, Macau SAR, China; lihuibo@bjmu.edu.cn (H.L.); whu@must.edu.mo (W.H.); 2109853dpp30001@student.must.edu.mo (Y.L.); 2 Department of Pharmacy, Peking University Third Hospital, Beijing 100191, China; wangjia11248@163.com (J.W.); huyang97@bjmu.edu.cn (Y.H.); 3 Institute for Drug Evaluation, Peking University Health Science Center, Beijing 100083, China; 4 Department of Pharmacy, Peking University Third Hospital Qinhuangdao Hospital, Qinhuangdao 066000, China; 5 Department of Pharmacy Administration and Clinical Pharmacy, School of Pharmaceutical Sciences, Peking University, Beijing 100871, China; 6 Department of Gastroenterology, Peking University Third Hospital, Beijing 100191, China; yanwt2003@163.com; 7 Shanghai Key Laboratory of Bioactive Small Molecules, Department of Pharmacology, School of Pharmacy, Fudan University, Shanghai 200437, China

**Keywords:** inflammatory bowel disease, biological product, small molecules, bibliometric study

## Abstract

**Objectives**: This bibliometric analysis investigates recent research trends in biologics and small molecules for treating inflammatory bowel disease (IBD) based on literature from the past decade. **Methods:** This cross-sectional study involved analyzing data retrieved from the Web of Science Core Collection (WoSCC) database to examine the evolution and thematic trends of biological agents and small-molecular drugs for IBD conducted between 1 January 2014, and 20 September 2024. VOSviewer software was utilized to assess co-authorship, co-occurrence, co-citation, and network visualization, followed by a further discussion on significant sub-themes. **Results:** From 2014 to 20 September 2024, the annual number of global publications increased by 23%, reflecting an acceleration in research activity. The journal “Inflammatory Bowel Diseases” published the highest number of manuscripts (579 publications) and garnered the most citations (13,632 citations), followed by the “Journal of Crohn’s & Colitis” (480 publications) and “Alimentary Pharmacology & Therapeutics” (250 publications). The United States led in productivity with 1943 publications and 66,320 citations, with UC San Diego (291) and authors Sandborn and Vermeire (180) topping the list. The co-occurrence cluster analysis of the top 100 keywords resulted in the formation of six distinct clusters: Disease Mechanisms, Drug Development, Surgical Interventions, Therapeutic Drug Monitoring (TDM), Immunological Targets, and Emerging Therapies. Burst terms (TNF-α inhibitors, JAK inhibitors, and trough-level optimization) highlight trends toward personalized biologics and small-molecule regimens. **Conclusions:** The bibliometric analysis indicates that IBD therapeutic research and clinical applications focus on biologics and small molecules, with research trends leaning toward precise therapy conversion or the combination in non-responders. Future work will assess monotherapy, the combination, and conversion therapies and investigate new drugs targeting inflammatory pathways.

## 1. Introduction

Inflammatory bowel disease (IBD) is a chronic inflammatory disorder of the gastrointestinal tract, distinct from typical acute gastroenteritis, primarily encompassing ulcerative colitis (UC), Crohn’s disease (CD), and indeterminate colitis [1]. Immune dysregulation is a critical factor in the pathogenesis of IBD [2]. IBD arises from a compromised intestinal barrier integrity, initiating T-cell-driven inflammation that is perpetuated by cytokine-mediated immune activation, culminating in chronic mucosal damage and complications such as intestinal fibrosis, strictures, abscesses, fistulas, malignancies, and extraintestinal manifestations [3,4,5,6].

**Figure 1:**
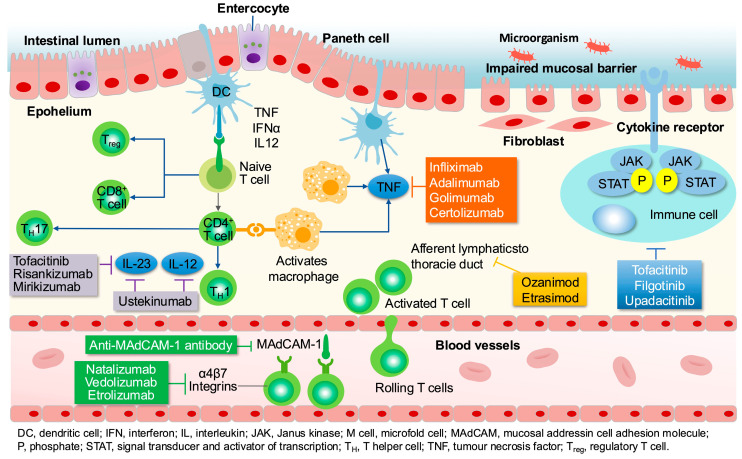
Targets and IBD biologics and small molecules for IBD. Under normal physiological conditions, T lymphocyte migration to the intestinal mucosa is tightly regulated. In pathological states, the integrity of the intestinal epithelial barrier is compromised, leading to increased permeability, allowing microorganisms or antigens to penetrate the intestinal wall, initially causing abnormal T lymphocyte migration, damaging the intestinal mucosa, and further exacerbating its permeability [13].

The primary goal in managing IBD is to relieve symptoms and reduce inflammation, thereby slowing disease progression and preventing complications [7]. Currently approved pharmacotherapies include traditional agents like mesalazine (a 5-aminosalicylic acid derivative), corticosteroids, and immunosuppressants such as azathioprine. Targeting lymphocyte migration to tackle inflammation caused by immune response dysfunction presents a promising therapeutic approach for IBD. Biologics, which include anti-tumor necrosis factor-alpha (TNF) agents, anti-interleukin IL12/23 inhibitors, and anti-integrin α4β7 monoclonal antibodies, along with small-molecule drugs like Janus kinase (JAK) inhibitors and S1P receptor modulators, function through this mechanism and are classified based on their specific targets [8,9,10,11,12] (Figure 1).

**Figure 2:**
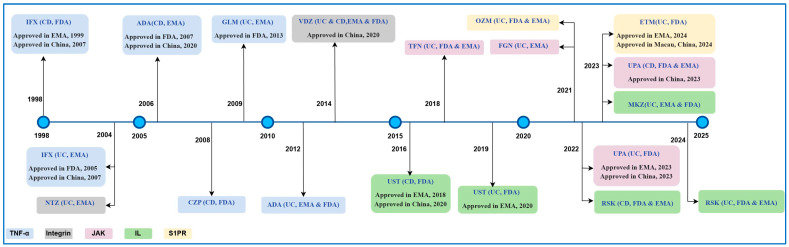
Global approval landscape for novel biologics and small molecules in IBD therapeutics. IFX (infliximab), ADA (adalimumab), CZP (certolizumab pegol), GLM (golimumab), VDZ (vedolizumab), NTZ (natalizumab), UST (ustekinumab), RSK (risankizumab), MKZ (mirikizumab), TFN (tofacitinib), FGN (filgotinib), UPA (upadacitinib), OZM (ozanimod), and ETM (etrasimod).

Up to 20% of patients with UC require a total colectomy, while 50% of those with CD undergo surgical intervention within ten years of diagnosis [14]. Currently, the surgical risk for individuals with IBD has decreased, likely due to ongoing improvements in pharmacological regimens, including new biologics and small molecules, along with advancements in endoscopic imaging technology [15,16]. Although many new drugs have been approved, their indications and approval timelines vary significantly. Most initial approvals occur in the United States and the European Union, followed by China and other developing countries. Figure 2 illustrates their approval timelines in recent years.

Bibliometric analysis has become an indispensable tool for quantifying research landscapes [17], enabling the systematic evaluation of academic outputs through temporal trend mapping, collaboration network visualization, and knowledge domain identification [18]. Particularly in rapidly evolving therapeutic fields like IBD [1], this methodology helps track paradigm shifts from traditional immunosuppressants to targeted biologics and small molecules—a transition that has fundamentally reshaped treatment since the 2014 approval of vedolizumab (the first gut-selective anti-integrin biologic) (Figure 2). While existing reviews focus on clinical efficacy or mechanistic classifications [7], there remains a striking paucity of quantitative, visualized analyses tracking how these scientific advances have shaped research priorities.

To address this gap, we employed bibliometric analysis to characterize the current state of research in these areas. Publications were sourced from the Web of Science Core Collection (WoSCC) covering the period from 2014 to 20 September 2024. This decadal analysis (2014–2024) serves as a Rosetta Stone for decoding how therapeutic innovation catalyzes research evolution, providing a methodological framework applicable to other fast-moving medical fields.

## 2. Results

### 2.1. The Trends in Annual Global Publications

**Figure 3:**
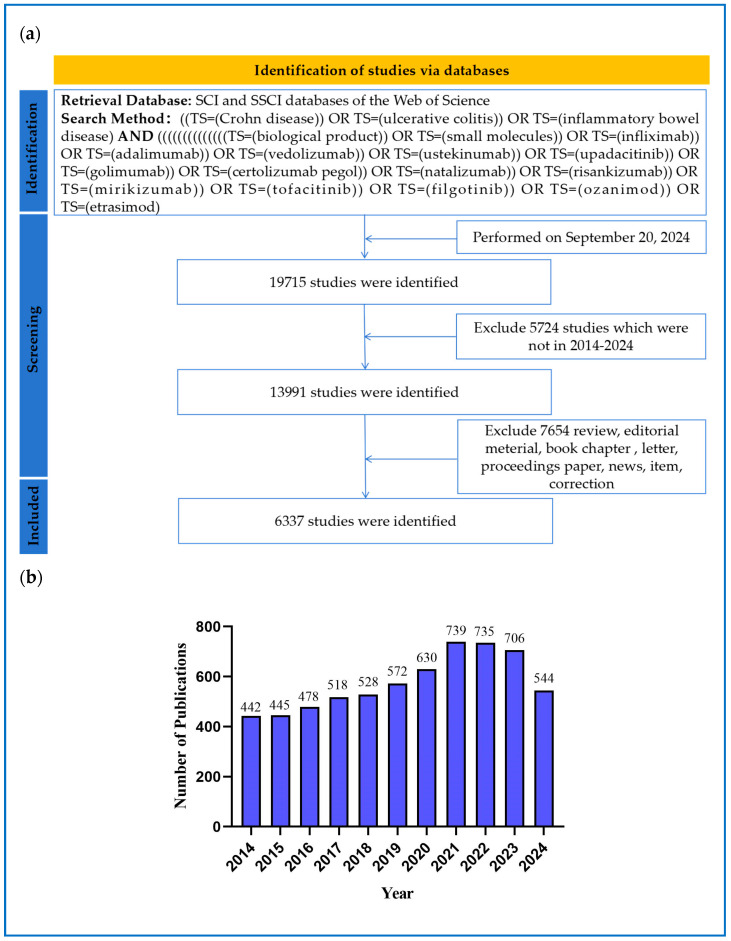
(**a**) The methodology for data collection and retrieval adheres to the PRISMA flowchart. (**b**) The number of articles regarding biologics and small molecules utilized in treating patients with IBD from 2014 to 2024.

As of our search date, 6337 articles related to biologics and small molecules for IBD were identified in the WoSCC according to the strategy for data collection and retrieval. The search results and screening process were systematically presented in accordance with the PRISMA flowchart guidelines to ensure the transparency and reproducibility of the study, as shown in Figure 3a. Figure 3b illustrates the number of articles published on the use of biologics and small molecules in treating patients with IBD from 2014 to 2024. The annual volume of publications serves as a critical metric for assessing the popularity and progression of research within a specific domain.

Overall, the volume of publications has exhibited an upward trend, peaking in 2021–2022, with a slight decline in 2023, yet still maintaining an overall upward trajectory. It remains uncertain whether 2024 will mark a turning point in research enthusiasm within this field. This analysis highlights the growing focus and significance that the global academic community places on biologics and small-molecule drugs for treating IBD.

### 2.2. Distribution of Source Journals

The articles on biologics or small molecules for IBD were published in 855 journals. Table 1 lists the top 10 journals that published the most articles on this topic, accounting for 36.7% (2326 out of 6337) of the total publications. “Inflammatory Bowel Diseases” emerged as the most prolific journal with 579 publications, followed by the “Journal of Crohn’s & Colitis” with 480 publications, and “Alimentary Pharmacology & Therapeutics”, with 250 publications.

### 2.3. Citation Analysis

A total of 6337 articles were cited 148,141 times. Table 2 presents the top 10 most cited articles on biologics and small molecules for IBD. The citation count for these top 10 articles ranges from 667 to 1386. Among these highly cited articles, two are European Crohn’s and Colitis Organisation (ECCO) guidelines, which are crucial for standardizing the clinical practices related to IBD treatment. Additionally, one article represents a European consensus that summarizes the collective insights and agreements among European experts in the field. The remaining seven articles are Randomized Controlled Trials (RCTs), recognized for providing high-level evidence in medical research. These top 10 articles were published in prestigious medical journals, including the New England Journal of Medicine, the Journal of Crohn’s and Colitis, The Lancet, and Gastroenterology.

### 2.4. Distribution and Co-Authorship of Countries/Regions

**Figure 4:**
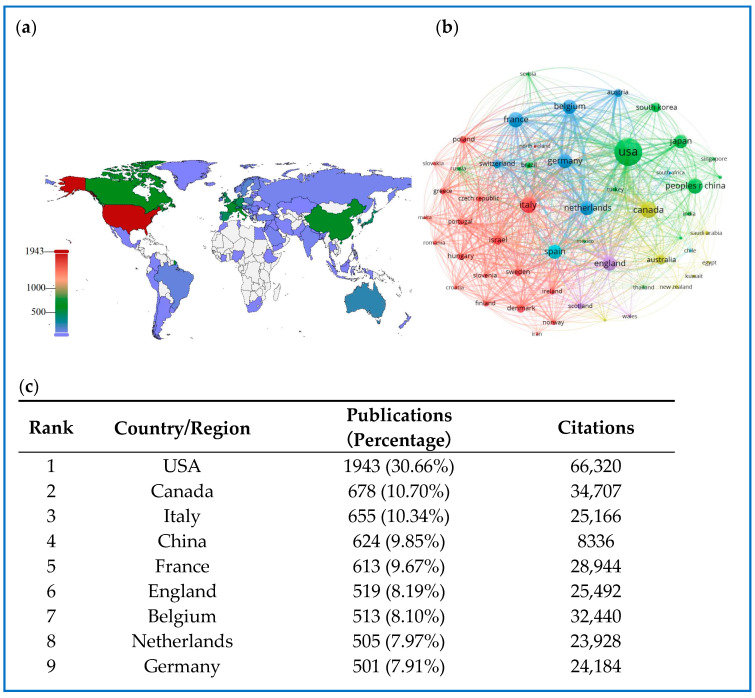
(**a**) Distribution of countries/regions. (**b**) The co-authorship network of countries/regions. (**c**) The top 10 productive countries/regions.

The exploration and therapeutic application of biologics and small molecules in treating IBD have attracted global attention, involving research contributions from 93 countries/regions, as illustrated in Figure 4a. The distribution map of research hotspots clearly indicates that North America, particularly the United States and Canada, and Western European countries such as Italy and France, exhibit significant interest in this research domain. Furthermore, China, with 6224 publications and 8336 citations, stands as a leading developing country in Asia, ranking fourth in scientific research contributions to this field, following the USA (1943 publications, 66,320 citations), Canada (678 publications, 34,707 citations), and Italy (655 publications, 25,166 citations).

Figure 4c illustrates other highly productive countries and regions ranked among the top 10 globally. A co-occurrence analysis of countries and territories was conducted using VOSviewer, revealing international collaboration networks within this field (Figure 4b). Of the 93 countries analyzed, 52 have published 10 or more articles, organized into six clusters, each represented by a distinct color. The largest cluster, shown in red, comprises 19 countries and is centered around Italy, Israel, and Denmark. The United States displayed the highest number of collaborative partners (n = 51), followed by Italy (n = 50), Canada (n = 49), the United Kingdom (n = 49), Germany (n = 47), France (n = 47), and the Netherlands (n = 47).

### 2.5. Distribution and Co-Authorship of Institutions

**Figure 5:**
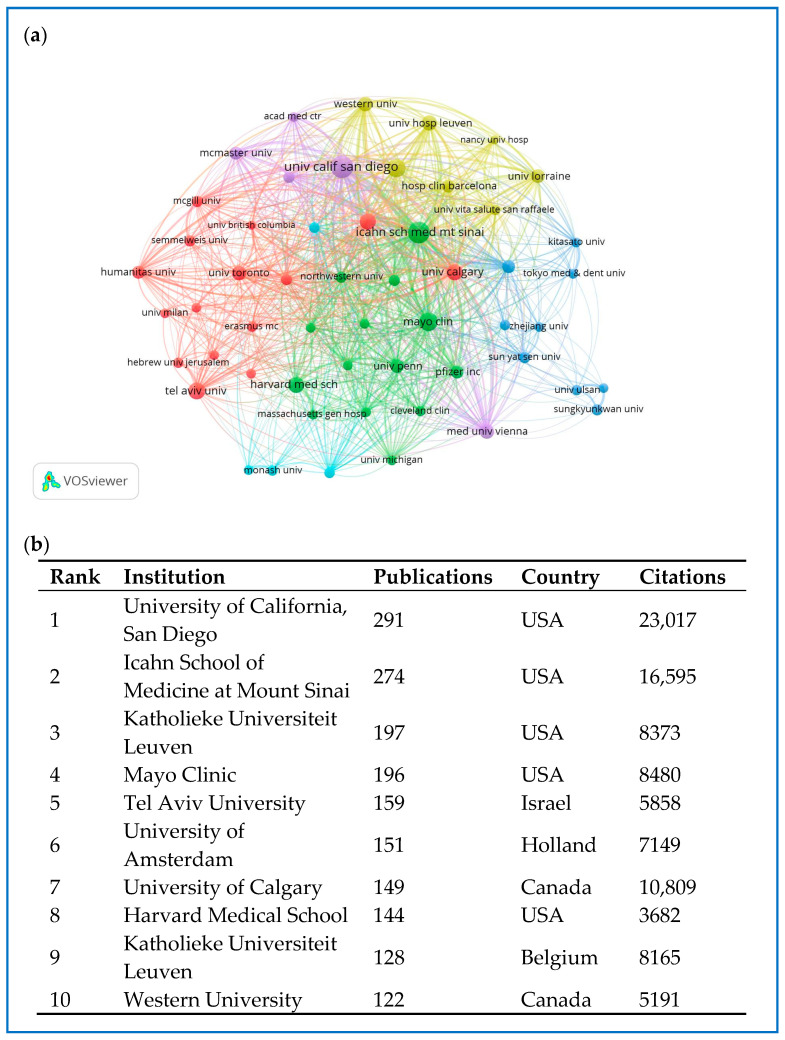
(**a**) The co-authorship network of institutions. (**b**) The top 10 productive institutions.

A total of 7430 institutions have contributed to research on biologics and small molecules for IBD. The top 10 most prolific institutions are shown in Figure 5b, with the University of California, San Diego leading (291 publications, 23,017 citations), followed by the Icahn School of Medicine at Mount Sinai (274 publications, 16,595 citations) and Katholieke Universiteit Leuven (197 publications, 8373 citations). After setting the threshold for the minimum number of published articles by institutions at 50, a total of 54 institutions met the criteria. VOSviewer was used to conduct a co-authorship analysis of these 54 prolific institutions (see Figure 5a). These institutions were categorized into six clusters, each represented by a unique color. The red cluster, consisting of 15 institutions, prominently included the University of Tel Aviv, the University of Amsterdam, and the University of Calgary, and was identified as the largest cluster. The Icahn School of Medicine at Mount Sinai had the highest number of collaborative partners (n = 50), followed by the Mayo Clinic (n = 48), the Medical University of Vienna (n = 48), and the University of Amsterdam (n = 47).

### 2.6. Distribution and Co-Authorship of Authors

**Figure 6:**
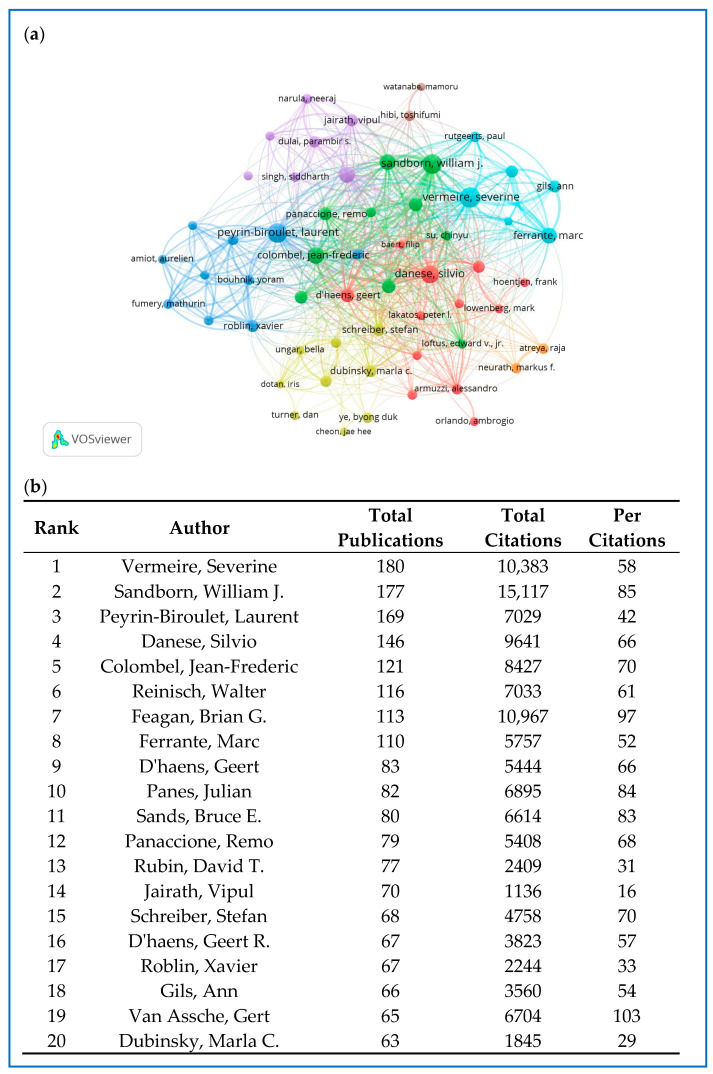
(**a**) The co-authorship network of authors. (**b**) The top 20 productive authors.

A total of 28,324 authors contributed to the publication of 6337 retrieved articles. Figure 6b shows the top 20 most productive authors. Vermeire, Severine, with 180 publications and 10,383 citations, emerged as the most prolific author, closely followed by Sandborn, William J, with 177 publications and 15,117 citations, and Peyrin-Billet, Laurent, with 169 publications and 7029 citations. In the current study, VOSviewer was used to perform a co-authorship analysis. A minimum threshold of 35 published articles per author was established. Among the 28,324 authors analyzed, 58 met this criterion. Figure 6a illustrates the co-authorship network of these authors, organized into eight clusters, each represented by a different color. The red cluster includes 12 authors, with Danese Silvio, D’haens Geert, and D’haens Geert R. at the center. Peyrin-Biroulet Laurent had the highest number of collaborative partners (n = 47), followed by Danese Silvio (n = 46), D’haens Geert (n = 46), and Vermeire Severine (n = 46).

### 2.7. Co-Citation Analysis

The 6337 publications retrieved cited 86,764 references, revealing a robust hierarchy of evidence in IBD therapeutics. Table 3 presents the top 10 co-cited references, with citations ranging from 349 to 817. Among these top 10 co-cited references, one is an editorial, while the others are all RCTs. Most of these RCTs are clinical studies centered on treating IBD with biologics. The foundational RCT “Infliximab for Induction and Maintenance Therapy for Ulcerative Colitis” (NEJM 2005) stands out as the cornerstone reference with 817 citations, highlighting its crucial role in establishing biologic therapy algorithms. Notably, six of these RCTs directly influenced FDA/EMA approval decisions (2005–2019), while three others (ranked #3, 5, and 9) became guideline-recommended comparators for newer agents.

### 2.8. The Co-Occurrence Analysis of the Top 100 Keywords

**Figure 7:**
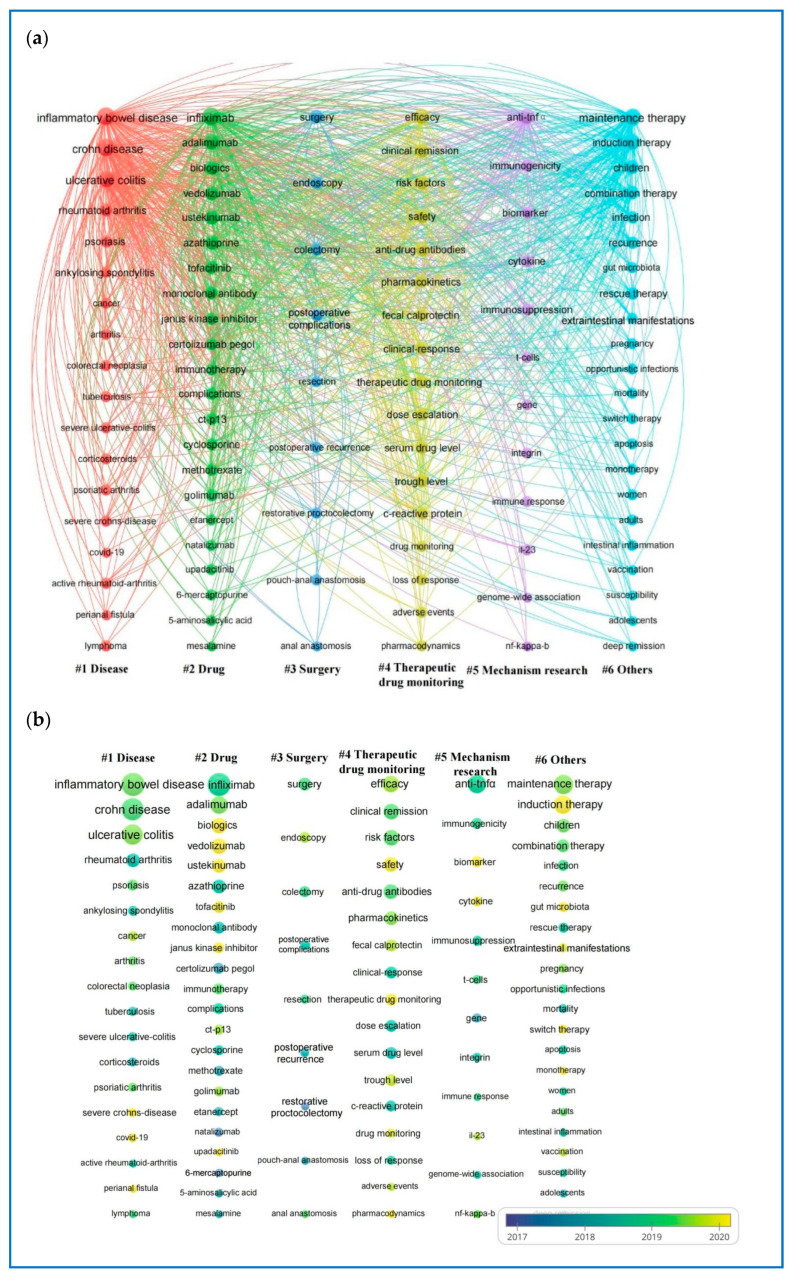
(**a**) The co-occurrence and cluster analysis of the top 100 keywords. (**b**) The overlay. Visualization of the top 100 keywords. Keywords with similar characteristics are organized into six clusters, each marked with a different color for visual distinction: red (Cluster 1—diseases), green (Cluster 2—medications), cerulean (Cluster 3—surgery), yellow (Cluster 4—therapeutic drug monitoring), purple (Cluster 5—IBD and drug mechanism research), and blue (Cluster 6—various topics such as populations, treatment types, complications, and precautions).

Keywords represent the main topics of publications, making high-frequency keywords especially suitable for co-occurrence analysis. In this study, VOSviewer extracted and clustered the top 100 keywords (see Supplementary Materials). Central to the network map are the following keywords: inflammatory bowel disease (3707 occurrences), infliximab (3305), Crohn’s disease (3205), ulcerative colitis (2267), maintenance therapy (1744), and anti-TNFα (1262). Figure 7a provides a visualization of the network map for these keywords, organized into six clusters based on their co-occurrence. The node labels represent the keywords, while the size of each node reflects the frequency of the keyword’s occurrence. Connections between nodes illustrate co-occurrence relationships among the relevant keywords.

To examine the temporal progression of research topic trends, keywords extracted from publications are visualized with a VOSviewer overlay and color-coded according to their average year of appearance (Figure 7b). Keywords depicted in cooler hues indicate those that emerged earlier, while those in warmer tones denote more recent occurrences. The most recent and commonly occurring keywords include biologics, vedolizumab, ustekinumab, tofacitinib, Janus kinase inhibitors, safety, therapeutic drug monitoring, biomarkers, cytokines, induction therapy, and gut microbiota.

## 3. Discussion

Our bibliometric analysis of the literature from the past decade has identified key research frontiers, hotspots, and challenges in clinical treatment. The results outlined in the previous section highlight the significant impact of biologics and small molecules on the treatment landscape of IBD. Detailed information on specific dosing regimens and therapeutic drug monitoring (TDM) strategies is presented in Table 4. In the upcoming discussion, we will examine the implications of these findings, propose strategies to address the identified issues, and explore ways to optimize current treatment plans.

### 3.1. Biologics for IBD

Although biologics are widely used in IBD management, approximately 30% of patients exhibit primary non-response, in which the treatment is initially ineffective. Furthermore, up to 50% of patients may experience a secondary loss of response, in which the treatment’s efficacy diminishes over time [25,26,27]. This loss of response is a complex phenomenon influenced by multiple factors.

The following are potential reasons for non-response: One possible reason for non-response is the significant heterogeneity of IBD, as patients exhibit diverse genetic profiles, immune responses, and disease phenotypes. If the primary causative factors are not effectively targeted by biological agents or involve other non-specific inflammatory pathways, this may result in suboptimal responses to specific biological therapies in certain individuals [28]. Secondly, inadequate drug dosing or incorrect dosing intervals, along with individual variations in drug metabolism and clearance rates, can influence the concentration of medications in the body and, subsequently, their therapeutic effectiveness. Thirdly, some biological agents are classified as humanized or chimeric monoclonal antibodies. Although these are designed to minimize immunogenicity, they may still provoke immune responses in patients. The production of anti-drug antibodies (ADAs) in a patient’s body can neutralize the drug and reduce its efficacy [29]. Fourthly, the prolonged use of biologics may lead to increased tolerance or adverse effects, contributing to a decrease in therapeutic efficacy. Potential side effects during biologic treatment include infections, malignancies, and immunogenic reactions, which could affect long-term treatment outcomes for patients [30].

**Figure 8:**
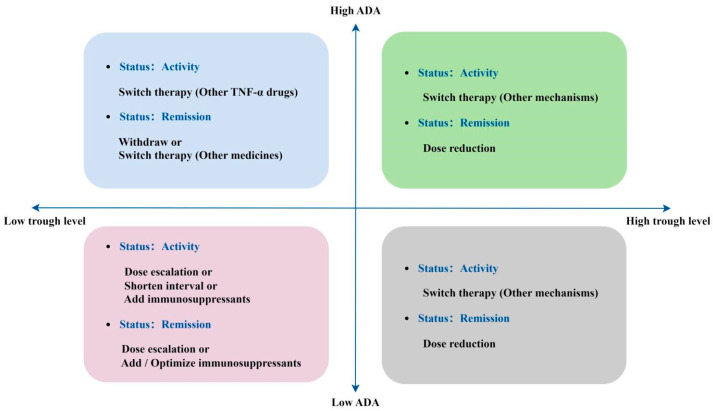
Summary treatment strategy adjustment of anti-TNF-α drugs.

In patients with a loss of response, in addition to immunosuppressants, TDM can be used to assess serum drug trough concentrations and anti-drug antibody (ADA) levels alongside the disease activity staging. This approach helps clinicians determine the need for medication adjustments, optimize treatment regimens, prevent adverse events, and enhance recovery outcomes [19,20,21,22,23,24] (Figure 8).

### 3.2. Small Molecules for IBD

Compared to biologics, small molecules such as JAK inhibitors and S1PR modulators are non-immunogenic, convenient for oral administration, possess a short half-life, exert rapid effects, and demonstrate a linear dose–response relationship. Additionally, they can serve as viable second-line treatment options for patients. However, the potential risks associated with these drugs, including thrombosis and infection, limit their clinical application. Therefore, it is crucial that we conduct comprehensive risk factor assessments—considering elements such as tumor presence, cardiovascular risk factors, age, and smoking habits—before selecting this class of medication. The FDA has clearly stated that upatinib may cause severe, potentially life-threatening adverse reactions, including serious infections, malignant tumors, major cardiovascular events, and thrombotic risks [31,32,33].

Despite these risks, medications are prescribed to treat various immune-mediated inflammatory diseases (IMIDs). When employing the same therapeutic strategy for different conditions, it is crucial that we adjust the dosage accordingly. Upatinib, the only JAK1 inhibitor approved for the treatment of IBD in China, is also indicated for other IMIDs such as atopic dermatitis, rheumatoid arthritis, psoriatic arthritis, and ankylosing spondylitis, with a recommended dosage of 15 mg once daily. However, for the induction phase of IBD treatment, the advised dosage is 45 mg once daily. Up to 50% of patients with IBD experience at least one extraintestinal manifestation [34], including peripheral arthritis, episcleritis, stomatitis, and erythema nodosum, which are often linked to IBD flare-ups. Additionally, conditions such as ankylosing spondylitis, sacroiliitis, uveitis, pyoderma gangrenosum, and primary sclerosing cholangitis are closely associated with IBD, though not necessarily related to its activity. These common extraintestinal manifestations fall under IMIDs [35]. Consequently, IBD patients presenting with extraintestinal symptoms require a multidisciplinary approach for diagnosis and treatment to determine the appropriate medication dosage, preventing ineffective therapy and disease progression.

S1PR modulators are associated with an increased risk of herpes zoster infection and transient cardiovascular events [31]. As dosages increase, the likelihood of adverse reactions and toxic side effects may also rise. The TDM of small molecules in IBD remains imperfect, and the AGA has not yet issued guidance on comparing the clinical efficacy and TDM of drugs with similar mechanisms [36,37,38].

### 3.3. Natural Herbal Medicine

Furthermore, drugs with potential therapeutic applications, such as berberine [39,40], Scutellaria baicalensis [41], curcumin [42], honeysuckle [43], and other traditional Chinese medicines derived from plants, have shown promising therapeutic effects on UC in animal studies. These traditional Chinese medicines have demonstrated effectiveness in modulating gut microbiota, managing inflammatory responses, repairing intestinal mucosa, and alleviating diarrhea. The associated signaling pathways include PI3K/Akt, NF-κB, JAK/STAT, MAPK, and Notch, among others. However, as many of these treatments are compound and decoction formulations, further research is needed to clarify the specific mechanisms of action and identify the primary active ingredients of individual compounds [42,44,45,46].

### 3.4. Limitations

While this bibliometric analysis provides new insights into the evolving landscape of IBD therapeutics, several limitations need attention. First, an inherent language bias is present since we exclusively analyzed English-language publications in WoSCC, potentially overlooking significant non-English studies—especially from East Asian and European regions where IBD research is expanding. Additionally, bibliometric methods naturally emphasize quantity over quality; while co-citation analysis highlights influential works, it cannot assess methodological rigor or clinical relevance. Future studies could triangulate findings with non-English databases (CNKI and LILACS) to mitigate these biases.

## 4. Materials and Methods

### 4.1. Data Source and Strategy for Retrieval

In this cross-sectional study, we implemented a comprehensive search strategy combining controlled vocabulary (e.g., MeSH terms, Bethesda, MD, USA) and free-text terms to maximize retrieval sensitivity. All data were collected from the Science Citation Index (SCI, Houston, TX, USA) and Social Sciences Citation Index (SSCI, Trondheim, Norway) databases of the Web of Science Core Collection (WoSCC, London, UK) on a single day (20 September 2024).

The search formula was constructed as follows:

TS = [(Crohn* disease “OR” ulcerative colitis “OR” inflammatory bowel disease “) AND (“biological product*” OR “small molecule*” OR “infliximab OR “adalimumab” OR “adalimumab” OR “vedolizumab” OR “ustekinumab” OR “upadacitinib” OR “golimumab” OR “determinolizumab pegol” OR “natalizumab” OR “riskizumab” OR “mirikizumab” OR “tofacitinib” OR “filmotinib”) OR “ozanimod” OR “etrasimod”].

The search lasted from January 2014 to 20 September 2024, and was restricted to “article” document types. The appendix details the specific data collection, validation process, rationale for drug name selection, and retrieval strategies (see Supplementary Materials).

### 4.2. Bibliometric Analysis and Visualization

By leveraging the technical tools and methodologies of information visualization, bibliometrics can effectively depict the research and development trajectory, current status, focal points, and emerging trends within a specific subject area [11]. Our methodology integrates triangulated analytical approaches, combining topological network analysis, temporal trend statistics, and semantic content mining. In this study, we utilized the online platform CNSknowal.com to create a distribution map that illustrates the countries and regions of publication for the articles analyzed [11,12]. Subsequently, using VOSviewer (version 1.6.20) [12], we conducted a visual analysis of authors, research institutions, countries/regions, citations, and keywords to explore the current research landscape and identify emerging hotspots within this academic domain.

## 5. Conclusions

Through bibliometric analysis and visualization techniques applied to keywords, our study reveals that biologics and small molecules are the primary focus of research and clinical applications among current therapeutic drugs for IBD. The immunogenicity of biologics and the safety concerns related to potential infections, thromboembolism, and the risk of malignant tumors associated with JAK inhibitors and S1P receptor modulators require significant attention.

The research emphasis has shifted from employing single-induction therapies and maintaining remission with various biologics and small-molecule drugs to precisely converting therapies or using drug combinations with differing mechanisms in cases of non-responsiveness. Future research efforts will focus on evaluating the efficacy and safety of current therapeutic drugs for monotherapy, combination therapy, and conversion therapy and developing new drugs targeting various inflammatory pathways.

## Figures and Tables

**Table 1:** The top 10 journals that published the most articles.

Rank	Source	Count (Percentage)	Citations(n)
1	Inflammatory Bowel Diseases	579 (9.14%)	13,632
2	Journal of Crohns & Colitis	480 (7.57%)	17,553
3	Alimentary Pharmacology & Therapeutics	250 (3.95%)	9681
4	Clinical Gastroenterology and Hepatology	178 (2.81%)	9260
5	Digestive Diseases and Sciences	177 (2.79%)	2202
6	Journal of Pediatric Gastroenterology and Nutrition	159 (2.51%)	2168
7	Scandinavian Journal of Gastroenterology	145 (2.29%)	1855
8	Digestive and Liver Disease	126 (1.99%)	1916
9	World Journal of Gastroenterology	117 (1.85%)	2439
10	European Journal of Gastroenterology & Hepatology	115 (1.81%)	1189

**Table 2:** The top 10 highest cited articles.

Rank	Title	Journal	Citations	PY	Doi	Type	Main Content
1	3rd European Evidence-based Consensus on the Diagnosis and Management of Crohn’s Disease 2016: Part 1: Diagnosis and Medical Management	J Crohns Colitis	1386	2017	10.1093/ecco-jcc/jjw168	Consensus	Incorporating the latest clinical practices and EMA/FDA certification documents for vedolizumab
2	Ustekinumab as Induction and Maintenance Therapy for Crohn’s Disease	N Engl J Med	1194	2016	10.1056/NEJMoa1602773	RCT	Systematically evaluated the efficacy and safety of ustekinumab as an induction and maintenance therapy for CD
3	Tofacitinib as Induction and Maintenance Therapy for Ulcerative Colitis	N Engl J Med	1121	2017	10.1056/NEJMc1707500	RCT	Tofacitinib is more effective than placebo in inducing and maintaining treatment of moderate to severe active ulcerative colitis
4	ECCO Guidelines on Therapeutics in Crohn’s Disease: Medical Treatment	J Crohns Colitis	785	2020	10.1093/ecco-jcc/jjz180	Guidelines	Identified research gaps in current treatment, such as the long-term safety of biologics and biomarkers for personalized therapy
5	Consensus guidelines of ECCO/ESPGHAN on the medical management of pediatric Crohn’s disease	J Crohns Colitis	737	2014	10.1016/j.crohns.2014.04.005	Guidelines	Internal medicine treatment, individualized treatment, and long-term management of CD in children and adolescents
6	Expanded allogeneic adipose-derived mesenchymal stem cells (Cx601) for complex perianal fistulas in Crohn’s disease: a phase 3 randomised, double-blind controlled trial	Lancet	706	2016	10.1016/S0140-6736(16)31203-X	RCT	Provides strong evidence of the effectiveness and safety of Cx601 in the treatment of complex perianal fistulas in CD
7	Ustekinumab as Induction and Maintenance Therapy for Ulcerative Colitis	N Engl J Med	702	2019	10.1056/NEJMoa1900750	RCT	Ustekinumab is effective in inducing and maintaining treatment of moderate to severe UC
8	Combination Therapy With Infliximab and Azathioprine Is Superior to Monotherapy With Either Agent in Ulcerative Colitis	Gastroenterology	677	2014	10.1053/j.gastro.2013.10.052	RCT	The efficacy and safety of IFX and AZA monotherapy and combination therapy in patients with moderate to severe UC were compared for the first time
9	Through Concentrations of Infliximab Guide Dosing for Patients With Inflammatory Bowel Disease	Gastroenterology	667	2015	10.1053/j.gastro.2015.02.031	RCT	Adjusting the dosage to achieve a trough concentration of 3–7 mg/mL of infliximab can significantly improve the remission rate of patients.
10	Subcutaneous golimumab induces clinical response and remission in patients with moderate-to-severe ulcerative colitis	Gastroenterology	629	2014	10.1053/j.gastro.2013.05.048	RCT	Systematically evaluated the induction therapy efficacy of golimumab in UC patients

**Table 3:** The top 10 most co-cited references.

Rank	Year	Journal	Title	Citations	Doi	Type	Main Content
1	2005	N Engl J Med	Infliximab for induction and maintenance therapy for ulcerative colitis	817	10.1056/nejmoa050516	RCT	The therapeutic potential of TNF—α in UC has been validated through large-scale clinical trials
2	2002	Lancet	Maintenance infliximab for Crohn’s disease: the ACCENT I randomized trial	801	10.1016/s0140-6736(02)08512-4	RCT	Large-scale clinical trials have validated the efficacy and safety of infliximab in long-term maintenance therapy for CD
3	2010	N Engl J Med	Infliximab, azathioprine, or combination therapy for Crohn’s disease	785	10.1056/nejmoa0904492	RCT	Evaluated the efficacy and safety of the combination therapy of infliximab and azathioprine in the treatment of CD
4	2013	N Engl J Med	Vedolizumab as induction and maintenance therapy for ulcerative colitis	644	10.1056/nejmoa1215734	RCT	Evaluated the induction and maintenance therapeutic effects of vedolizumab in UC
5	2013	N Engl J Med	Vedolizumab as induction and maintenance therapy for Crohn’s disease	596	10.1056/nejmoa1215739	RCT	Evaluated the induction and maintenance therapeutic effects of vedolizumab in CD
6	2016	Gastroenterology	Adalimumab for maintenance of clinical response and remission in patients with Crohn’s disease: the CHARM trial	510	10.1053/j.gastro.2006.11.041	RCT	Adalimumab was proven for the first time to be effective and safe in maintaining long-term remission in CD
7	2016	N Engl J Med	Ustekinumab as Induction and Maintenance Therapy for Crohn’s Disease	437	10.1056/nejmoa1602773	RCT	Proving the induction and maintenance therapeutic effects of ustekinumab in CD
8	2011	Gastroenterology	Adalimumab induces and maintains clinical remission in patients with moderate-to-severe ulcerative colitis	361	10.1053/j.gastro.2011.10.032	RCT	Identifying the induction and maintenance therapeutic effects of adalimumab in moderate to severe UC
9	2005	Gut	The Montreal classification of inflammatory bowel disease: controversies, consensus, and implications	357	10.1136/gut.2005.082909	Leading Article	Reviewing the classification of IBD
10	1987	N Engl J Med	Coated oral 5-aminosalicylic acid therapy for mildly to moderately active ulcerative colitis. A randomized study	349	10.1056/nejm198712243172603	RCT	The efficacy of 5-ASA in mild to moderate UC was proven for the first time

**Table 4:** Administration strategies and trough level of approved biologics and small molecules for IBD.

Drug	Target	Indication	Induction Therapy		Maintenance Therapy		Trough Level (μg/mL)
			Dose	By	Dose	By	
infliximab	TNF-α	UC, CD	5 mg/kg at 0, 2, 6 wk	iv	5 mg/kg q8w	iv	≥5 [19]; 3–7 [20]; 5–10 [21]; 7–10 (I); 5–7 (M) [22]
adalimumab	TNF-α	UC, CD	160 mg day 1, 80 mg day 15	sc	40 mg q2w	sc	≥7.5 [19]; 4–8 [20]; 8–12 [21]; >10–12 (I,M) [22]
golimumab	TNF-α	UC	200 mg day 1, 100 mg day 15	sc	100 mg q4 w; <80 kg, 50 mg q4w; >80 kg, 100 mg q4w	sc	>2.5 [23]
certolizumab	TNF-α	CD	400 mg 0, 2, 4 wk	sc	400 mg q4w	sc	≥20 [19]
vedolizumab	α4β7 Integrin	UC, CD	300 mg at 0, 2, 6 wk	iv	300 mg q8w	iv	>33–37 (6 wk); >15–20 (14 wk); >10–15 (M) [24]
natalizumab	α4 Integrin	CD	300 mg at 0, 4, 8, 12 wk	iv	300 mg, q4w	iv	
ustekinumab	IL-12/23 p40	UC, CD	<55 kg, 260 mg 55–85 kg, 390 mg >85 kg, 520 mg	iv	90 mg q8w	sc	>3–7 (8 wk); >1–3 (M) [24]; >11.1 (I); >4.5 (M) [22]
risankizumab	IL-23p19	CD	600 mg at 0, 4, 8 wk	iv	180 mg or 360 mg q8w	sc	NA
mirikizumab	IL-23p19	UC	300 mg at 0, 4, 8 wk	iv	200 mg at 12w, q4w	iv	
tofacitinib	JAK-1/2/3	UC	10 mg bid for 8 weeks	po	5 mg or 10 mg bid	po	
upadacitinib	JAK-1	UC, CD	45 mg daily for 8 weeks (UC) 12 weeks (CD)	po	15 mg or 30 mg qd	po	
filgotinib	JAK-1	UC	200 mg qd for 22 weeks	po	200 mg qd	po	
ozanimod	S1PR	UC	0.23 mg qd, day 1–4 0.46 mg qd, day 5–7	po	0.92 mg qd	po	
etrasimod	S1PR	UC	2 mg qd	po	2 mg qd	po	

I (Induction), and M (Maintenance).

## Data Availability

The original contributions presented in this study are included in the article/Supplementary Materials. Further inquiries can be directed to the corresponding authors.

## Supplementary Materials

The following supporting information can be downloaded at: https://www.mdpi.com/article/10.3390/ph18030312/s1, Search strategy; Table S1: Clusters of the top 100 keywords.
