# Supplementary material for: Mapping the Evolution of IBD Treatment: A Bibliometric Study on Biologics and Small Molecules
Source: Pharmaceuticals (Basel). 2025 Feb 24;18(3):312. doi: 10.3390/ph18030312 (PMC11944940; doi:10.3390/ph18030312)
Supplement: Supplementary file 1 [file pharmaceuticals-18-00312-s001.zip › pharmaceuticals-3456469-supplementary.pdf]

Supplementary Material

1. Search strategy:

#1 'Crohn disease'[Mesh] OR 'cleron disease' OR 'Crohn`s disease' OR 'Crohns disease' OR 'enteritis regionalis' OR 'intestinal tract, regional enteritis' OR 'morbus crohn' OR 'regional enteritis' OR 'regional enterocolitis'

#2 'ulcerative colitis'[Mesh] OR 'colitis ulcerativa' OR 'colitis ulcerosa' OR 'colitis ulcerosa chronica' OR 'colitis, mucosal' OR 'colitis, ulcerative' OR 'colitis, ulcerous' OR 'colon, chronic ulceration' OR 'histiocytic ulcerative colitis' OR 'mucosal colitis' OR 'ulcerative colorectitis' OR 'ulcerative procto colitis' OR 'ulcerative proctocolitis' OR 'ulcerous colitis' OR 'ulcerative colitis'

#3 'inflammatory bowel disease'[Mesh] OR 'inflammatory bowel diseases'

#4= #1 or #2 or #3

#5 'biological product'[Mesh] OR 'biologic' OR 'biologic agent' OR 'biologic agents' OR 'biologic product' OR 'biologic products' OR 'biological' OR 'biological agent' OR 'biological agents' OR 'biological products' OR 'biologicals' OR 'biologics'

#6 'infliximab'[Mesh] OR 'abp 710' OR 'abp710' OR 'avakine' OR 'avsola' OR 'bcd 055' OR 'bcd055' OR 'bow 015' OR 'bow015' OR 'cmab 008' OR 'cmab008' OR 'ct p13' OR 'ctp 13' OR 'ctp13' OR 'flammegis' OR 'flixabi' OR 'gb 242' OR 'gb242' OR 'gp 1111' OR 'gp1111' OR 'infimab' OR 'inflectra' OR 'infliximab abda' OR 'infliximab axxq' OR 'infliximab dyyb' OR 'infliximab hjmt' OR 'infliximab qbtx' OR 'infliximab-abda' OR 'infliximab-axxq' OR 'infliximab-dyyb' OR 'infliximab-hjmt' OR 'infliximab-qbtx' OR 'ixifi' OR 'ni 071' OR 'ni071' OR 'pf 06438179' OR 'pf 6438179' OR 'pf06438179' OR 'pf6438179' OR 'remicade' OR 'remsima' OR 'renflexis' OR 'revellex' OR 'ro 6897845' OR 'ro6897845' OR 'sb 2' OR 'sb2' OR 'sti 002' OR 'sti002' OR 'ta 650' OR 'ta650' OR 'zessly' OR 'zymfentra'

#7 'adalimumab'[Mesh] OR 'abp 501' OR 'abp501' OR 'abrilada' OR 'abt d2e7' OR 'abtd2e7' OR 'adalacip' OR 'adalimumab aacf' OR 'adalimumab aaty' OR 'adalimumab adaz' OR 'adalimumab adbm' OR 'adalimumab afzb' OR 'adalimumab aqvh' OR 'adalimumab atto' OR 'adalimumab beta' OR 'adalimumab bwwd' OR 'adalimumab eva' OR 'adalimumab fkjp' OR 'adalimumab ryvk' OR 'adalimumab-aacf' OR 'adalimumab-aaty' OR 'adalimumab-adaz' OR 'adalimumab-adbm' OR 'adalimumab-afzb' OR 'adalimumab-aqvh' OR 'adalimumab-atto' OR 'adalimumab-bwwd' OR 'adalimumab-eva' OR 'adalimumab-fkjp' OR 'adalimumab-ryvk' OR 'adalloce' OR 'adaly' OR 'amgevita' OR 'amjevita' OR 'amsparity' OR 'ardalicip' OR 'avt 02' OR 'avt02' OR 'bat 1406' OR 'bat1406' OR 'bax 2923' OR 'bax 923' OR 'bax2923' OR 'bax923' OR 'bcd 057' OR 'bcd057' OR 'bi 695501' OR 'bi695501' OR 'bmo 2' OR 'bmo2' OR 'bxt 2922' OR 'bxt2922' OR 'chs 1420' OR 'chs1420' OR 'cinnora' OR 'ciptunec' OR 'ct p17' OR 'ctp17' OR 'cyltezo' OR 'da 3113' OR 'da3113' OR 'dalibra' OR 'dmb 3113' OR 'dmb3113' OR 'euplima' OR 'exemptia' OR 'fkb 327' OR 'fkb327' OR 'fyzoclad' OR 'gp 2017' OR 'gp2017' OR 'hadlima' OR 'halimatoz' OR 'hefiya' OR 'hlx 03' OR 'hlx03' OR 'hs 016' OR 'hs016' OR 'hukyndra' OR 'hulio' OR 'humira' OR 'hyrimoz' OR 'ibi 303' OR 'ibi303' OR 'idacio' OR 'imraldi' OR 'jy 026' OR 'jy026' OR 'kromeya' OR 'libmyris' OR 'lu 200134' OR 'lu200134' OR 'm 923' OR 'm923' OR 'mabura' OR 'monoclonal antibody D2E7' OR 'msb 11022' OR 'msb11022' OR 'myl 1401a' OR 'myl1401a' OR 'ons 3010' OR 'ons3010' OR 'pbp 1502' OR 'pbp1502' OR 'pf 06410293' OR 'pf 6410293' OR 'pf06410293' OR 'pf6410293' OR 'qletli' OR 'raheara' OR 'sb 5' OR 'sb5' OR 'simlandi' OR 'solymbic' OR 'sulinno' OR 'trudexa' OR 'tur 01' OR 'tur01' OR 'uplima' OR 'yuflyma' OR 'yusimry' OR 'zrc 3197' OR 'zrc3197'

#8 'vedolizumab'[Mesh] OR 'entyvio' OR 'kynteles' OR 'ldp 02' OR 'ldp02' OR 'mln 0002' OR 'mln 02' OR 'mln 02 antibody' OR 'mln 02 monoclonal antibody' OR 'mln0002' OR 'mln02' OR 'mln02 antibody' OR 'mln02 monoclonal antibody' OR 'monoclonal antibody ldp 02' OR 'monoclonal antibody mln 02' OR 'pb 016' OR 'pb016' OR 'ro 7246311' OR 'ro7246311'

#9 'ustekinumab'[Mesh] OR 'abp 654' OR 'abp654' OR 'amg 654' OR 'amg654' OR 'avt 04' OR 'avt04' OR 'bat 2206' OR 'bat2206' OR 'bfi 751' OR 'bfi751' OR 'bmab 1200' OR 'bmab1200' OR 'bow 090' OR 'bow090' OR 'cnto 1275' OR 'cnto1275' OR 'ct p43' OR 'ctp43' OR 'da 3115' OR 'da3115' OR 'dmb 3115' OR 'dmb3115' OR 'eb 1004' OR 'eb1004' OR 'fyb 202' OR 'fyb202' OR 'jamteki' OR 'monoclonal antibody cnto 1275' OR 'ons 3040' OR 'ons3040' OR 'pb 007' OR 'pb007' OR 'pyzchiva' OR 'ro 723 3920' OR 'ro 7233920' OR 'ro7233920' OR 'sb 17' OR 'sb17' OR 'selarsdi' OR 'stelara' OR 'stellara' OR 'suterara' OR 'tt 20' OR 'tt20' OR 'ustekinumab aekn' OR 'ustekinumab auub' OR 'ustekinumab-aekn' OR 'ustekinumab-auub' OR 'uzpruvo' OR 'wezenla' OR 'wezlana'

#10 'upadacitinib'[Mesh] OR '3 ethyl 4 (1, 5, 7, 10 tetrazatricyclo [7.3.0.0 (2, 6)] dodeca 2 (6), 3, 7, 9, 11 pentaen 12 yl) n (2, 2, 2 trifluoroethyl) 1 pyrrolidinecarboxamide' OR '3 ethyl 4 (1, 5, 7, 10 tetrazatricyclo [7.3.0.0 (2, 6)] dodeca 2 (6), 3, 7, 9, 11 pentaen 12 yl) n (2, 2, 2 trifluoroethyl) pyrrolidine 1 carboxamide' OR '3 ethyl 4 (3h imidazo [1, 2 a] pyrrolo [2, 3 e] pyrazin 8 yl) n (2, 2, 2 trifluoroethyl) 1 pyrrolidinecarboxamide' OR '3 ethyl 4 (3h imidazo [1, 2 a] pyrrolo [2, 3 e] pyrazin 8 yl) n (2, 2, 2 trifluoroethyl) 1 pyrrolidinecarboxamide 2, 3 dihydroxybutanedioate' OR '3 ethyl 4 (3h imidazo [1, 2 a] pyrrolo [2, 3 e] pyrazin 8 yl) n (2, 2, 2 trifluoroethyl) 1 pyrrolidinecarboxamide tartrate' OR '3 ethyl 4 (3h imidazo [1, 2 a] pyrrolo [2, 3 e] pyrazin 8 yl) n (2, 2, 2 trifluoroethyl) pyrrolidine 1 carboxamide' OR '3 ethyl 4 (3h imidazo [1, 2 a] pyrrolo [2, 3 e] pyrazin 8 yl) n (2, 2, 2 trifluoroethyl) pyrrolidine 1 carboxamide 2, 3 dihydroxybutanedioate' OR '3 ethyl 4 (3h imidazo [1, 2 a] pyrrolo [2, 3 e] pyrazin 8 yl) n (2, 2, 2 trifluoroethyl) pyrrolidine 1 carboxamide tartrate' OR '3 ethyl 4 (imidazo [1, 2 a] pyrrolo [2, 3 e] pyrazin 8 yl) n (2, 2, 2 trifluoroethyl) 1 pyrrolidinecarboxamide' OR '3 ethyl 4 (imidazo [1, 2 a] pyrrolo [2, 3 e] pyrazin 8 yl) n (2, 2, 2 trifluoroethyl) pyrrolidine 1 carboxamide' OR 'abt 494' OR 'abt494' OR 'rinvoq' OR 'rinvoq lq' OR 'upadacitinib 2, 3 dihydroxybutanedioate' OR 'upadacitinib hemihydrate' OR 'upadacitinib hydrate' OR 'upadacitinib tartrate'

#11 'golimumab'[Mesh] OR 'cnto 148' OR 'cnto148' OR 'mk 8259' OR 'mk8259' OR 'sch 900259' OR 'sch900259' OR 'shinponi' OR 'simponi' OR 'simponi aria'

#12 'certolizumab pegol'[Mesh] OR 'cdp 870' OR 'cdp870' OR 'cimzia' OR 'pegylated tumor necrosis factor alpha antibody Fab fragment' OR 'pegylated tumour necrosis factor alpha antibody Fab fragment' OR 'pf 688' OR 'pf688' OR 'pha 738144' OR 'pha738144' OR 'simziya' OR 'xcimzane'

#13 'natalizumab'[Mesh] OR 'an 10022' OR 'an 100226' OR 'an10022' OR 'an100226' OR 'antegran' OR 'antegren' OR 'bg 0002' OR 'bg0002' OR 'dst 356a1' OR 'dst356a1' OR 'natalizumab sztn' OR 'natalizumab-sztn' OR 'pb 006' OR 'pb006' OR 'pbp 2002' OR 'pbp2002' OR 'tyruko' OR 'tysabri'

#14 'risankizumab'[Mesh] OR 'abbv 066' OR 'abbv066' OR 'bi 655066' OR 'bi655066' OR 'risankizumab rzaa' OR 'risankizumab-rzaa' OR 'skyrizi'

#15 'mirikizumab'[Mesh] OR 'ly 3074828' OR 'ly3074828' OR 'mirikizumab mrkz' OR 'mirikizumab-mrkz' OR 'omvoh'

#16 'tofacitinib'[Mesh] OR '1 cyanoacetyl 4 methyl n methyl n (1 hydropyrrolo [2, 3 d] pyrimidin

4 yl) 3 piperidinamine' OR '1 cyanoacetyl 4 methyl n methyl n (1 hydropyrrolo [2, 3 d] pyrimidin 4 yl) piperidine 3 amine' OR '1 cyanoacetyl 4 methyl n methyl n (1h pyrrolo [2, 3 d] pyrimidin 4 yl) 3 piperidinamine' OR '1 cyanoacetyl 4 methyl n methyl n (1h pyrrolo [2, 3 d] pyrimidin 4 yl) piperidine 3 amine' OR '3 [4 methyl 3 [methyl (7 hydropyrrolo [2, 3 d] pyrimidin 4 yl) amino] 1 piperidinyl] 3 oxopropanenitrile' OR '3 [4 methyl 3 [methyl (7 hydropyrrolo [2, 3 d] pyrimidin 4 yl) amino] 1 piperidyl] 3 oxopropanenitrile' OR '3 [4 methyl 3 [methyl (7 hydropyrrolo [2, 3 d] pyrimidin 4 yl) amino] piperidin 1 yl] 3 oxopropanenitrile' OR '3 [4 methyl 3 [methyl (7h pyrrolo [2, 3 d] pyrimidin 4 yl) amino] 1 piperidinyl] 3 oxopropanenitrile' OR '3 [4 methyl 3 [methyl (7h pyrrolo [2, 3 d] pyrimidin 4 yl) amino] 1 piperidyl] 3 oxopropanenitrile' OR '3 [4 methyl 3 [methyl (7h pyrrolo [2, 3 d] pyrimidin 4 yl) amino] piperidin 1 yl] 3 oxopropanenitrile' OR '4 [n [1 (2 cyano 1 oxoethyl) 4 methyl 3 piperidinyl] n methylamino] pyrrolo [2, 3 d] pyrimidine' OR '4 [n [1 (2 cyano 1 oxoethyl) 4 methyl 3 piperidyl] n methylamino] pyrrolo [2, 3 d] pyrimidine' OR '4 methyl 3 [methyl (7h pyrrolo [2, 3 d] pyrimidin 4 yl) amino] beta oxo 1 piperidinepropanenitrile' OR 'cgb 500' OR 'cgb500' OR 'cp 690 550' OR 'cp 690, 550' OR 'cp 690550' OR 'cp 690550 10' OR 'cp 690550-10' OR 'cp690 550' OR 'cp690, 550' OR 'cp690550' OR 'cp690550 10' OR 'cp690550-10' OR 'jaquinus' OR 'pgn 600' OR 'pgn600' OR 'prd 4862257' OR 'prd4862257' OR 'ro 5169503' OR 'ro5169503' OR 'tasocitinib' OR 'tasocitinib citrate' OR 'tofacitinib citrate' OR 'xeljanz' OR 'xeljanz xr'

#17 'filgotinib'[Mesh] OR 'filgotinib 2 butenedioate' OR 'filgotinib hydrochloride' OR 'filgotinib maleate' OR 'g 146034' OR 'g 146034 101' OR 'g 146034-101' OR 'g146034' OR 'g146034 101' OR 'g146034-101' OR 'glpg 0634' OR 'glpg0634' OR 'gs 6034' OR 'gs6034' OR 'jyseleca' OR 'n [5 [4 (1, 1 dioxothiomorpholinomethyl) phenyl] 1, 2, 4 triazolo [1, 5 a] pyridin 2 yl] cyclopropanecarboxamide' OR 'n [5 [4 (1, 1 dioxothiomorpholinomethyl) phenyl] 1, 2, 4 triazolo [1, 5 a] pyridin 2 yl] cyclopropanecarboxamide 2 butenedioate' OR 'n [5 [4 (1, 1 dioxothiomorpholinomethyl) phenyl] 1, 2, 4 triazolo [1, 5 a] pyridin 2 yl] cyclopropanecarboxamide but 2 enedioate' OR 'n [5 [4 [(1, 1 dioxido 4 thiomorpholinyl) methyl] phenyl] 1, 2, 4 triazolo [1, 5 a] pyridin 2 yl] cyclopropanecarboxamide' OR 'n [5 [4 [(1, 1 dioxido 4 thiomorpholinyl) methyl] phenyl] 1, 2, 4 triazolo [1, 5 a] pyridin 2 yl] cyclopropanecarboxamide 2 butenedioate' OR 'n [5 [4 [(1, 1 dioxidothiomorpholin 4 yl) methyl] phenyl] [1, 2, 4] triazolo [1, 5 a] pyridin 2 yl] cyclopropanecarboxamide' OR 'n [5 [4 [(1, 1 dioxo 1, 4 thiazinan 4 yl) methyl] phenyl] [1, 2, 4] triazolo [1, 5 a] pyridin 2 yl] cyclopropanecarboxamide' OR 'n [5 [4 [(1, 1 dioxothiomorpholin 4 yl) methyl] phenyl] 1, 2, 4 triazolo [1, 5 a] pyridin 2 yl] cyclopropanecarboxamide' OR 'n [5 [4 [(1, 1 dioxothiomorpholin 4 yl) methyl] phenyl] 1, 2, 4 triazolo [1, 5 a] pyridin 2 yl] cyclopropanecarboxamide but 2 enedioate' OR 'n [5 [4 [(1, 1 dioxothiomorpholin 4 yl) methyl] phenyl] [1, 2, 4] triazolo [1, 5 a] pyridin 2 yl] cyclopropanecarboxamide' OR 'n [5 [4 [(1, 1 dioxothiomorpholin 4 yl) methyl] phenyl] [1, 2, 4] triazolo [1, 5 a] pyridin 2 yl] cyclopropanecarboxamide but 2 enedioate'

#18 'ozanimod'[Mesh] OR '5 [3 [1 [(2 hydroxyethyl) amino] 2, 3 dihydro 1h inden 4 yl] 1, 2, 4 oxadiazol 5 yl] 2 [(2 propanyl) oxy] benzonitrile' OR '5 [3 [1 [(2 hydroxyethyl) amino] 2, 3 dihydro 1h inden 4 yl] 1, 2, 4 oxadiazol 5 yl] 2 [(propan 2 yl) oxy] benzonitrile' OR '5 [3 [1 [(2 hydroxyethyl) amino] 2, 3 dihydro 1h inden 4 yl] 1, 2, 4 oxadiazol 5 yl] 2 isopropoxybenzonitrile' OR '5 [3 [1 [(2 hydroxyethyl) amino] 4 indanyl] 1, 2, 4 oxadiazol 5 yl] 2 isopropoxybenzonitrile' OR '5 [3 [2, 3 dihydro 1 [(2 hydroxyethyl) amino] 1h inden 4 yl] 1, 2, 4 oxadiazol 5 yl] 2 (1 methylethoxy) benzonitrile' OR '5 [3 [2, 3 dihydro 1 [(2 hydroxyethyl)

amino] 1h inden 4 yl] 1, 2, 4 oxadiazol 5 yl] 2 isopropoxybenzonitrile' OR 'ozanimod hydrochloride' OR 'rpc 1063' OR 'rpc1063' OR 'zeposia'

#19 'etrasimod'[Mesh] OR '2 [7 [[4 cyclopentyl 3 (trifluoromethyl) benzyl] oxy] 1, 2, 3, 4 tetrahydrocyclopenta [b] indol 3 yl] acetic acid' OR '2 [7 [[4 cyclopentyl 3 (trifluoromethyl) phenyl] methoxy] 1, 2, 3, 4 tetrahydrocyclopenta [b] indol 3 yl] acetic acid' OR '7 [[4 cyclopentyl 3 (trifluoromethyl) benzyl] oxy] 1, 2, 3, 4 tetrahydrocyclopent [b] indole 3 acetic acid' OR '7 [[4 cyclopentyl 3 (trifluoromethyl) phenyl] methoxy] 1, 2, 3, 4 tetrahydrocyclopent [b] indole 3 acetic acid' OR '7 [[4 cyclopentyl 3 (trifluoromethyl) phenyl] methoxy] 1, 2, 3, 4 tetrahydrocyclopent [b] indole 3 acetic acid arginine' OR '7 [[[4 cyclopentyl 3 (trifluoromethyl) benzyl] oxy] 1, 2, 3, 4 tetrahydrocyclopenta [b] indol 3 yl] acetic acid' OR '7 [[[4 cyclopentyl 3 (trifluoromethyl) phenyl] methoxy] 1, 2, 3, 4 tetrahydrocyclopenta [b] indol 3 yl] acetic acid' OR '[7 [[4 cyclopentyl 3 (trifluoromethyl) benzyl] oxy] 1, 2, 3, 4 tetrahydrocyclopenta [b] indol 3 yl] acetic acid' OR '[7 [[4 cyclopentyl 3 (trifluoromethyl) phenyl] methoxy] 1, 2, 3, 4 tetrahydrocyclopenta [b] indol 3 yl] acetic acid' OR 'apd 334' OR 'apd334' OR 'arginine mono [7[[4 cyclopentyl 3 (trifluoromethyl) phenyl] methoxy] 1, 2, 3, 4 tetrahydrocyclopenta [b] indol 3 yl] acetic acid' OR 'etrasimod arginine' OR 'pf 07915503' OR 'pf 7915503' OR 'pf07915503' OR 'pf7915503' OR 'velsipity'

#20=#5 or #6 or #7 or #8 or #9 or #10 or #11 or #12 or #13 or #14 or #15 or #16 or #17 or #18 or #19

#21=#4 and #20

(1) The search terms were determined by the #21

(2) The document type was “article”;

(3) The publication period was between 2014 and 2024.

(4) The above information was collected: publication, authors, countries, institutions, journals, keywords, and citations.

2. The parameters for using VOS visualization mapping

Multidimensional Network Modeling Using VOSviewer 1.6.20, we constructed:

Co-authorship of countries/regions: Threshold = minimum 10, Normalization Method: Linlog/modularity, Layout Attraction:2, Layout Repulsion: -1, Clustering Resolution 1.00, Min. cluster size 1.

Co-authorship of institutions: Threshold = minimum 50, Normalization Method: Association strength, Layout Attraction: 0, Layout Repulsion: -3, Clustering Resolution 1.00, Min. cluster size 2.

Co-authorship of authors: Threshold = minimum 35, Normalization Method: Association strength, Layout Attraction: 1, Layout Repulsion: -4, Clustering Resolution 1.00, Min. cluster size 2.

Co-occurrence of Keywords: After merging and counting the occurrence of synonymous keywords, select the top 100 keywords for visual imaging and divide them into 6 clusters.

Table S1. Clusters of the top 100 Keywords

Cluster	Keywords	Counts	Rank	Cluster	Keywords	Counts	Rank
1	inflammatory bowel disease	3707	1	4	clinical remission	729	10
1	crohn disease	3205	3	4	risk factors	611	13
1	ulcerative colitis	2267	4	4	safety	581	14
1	rheumatoid arthritis	437	18	4	anti-drug antibodies	473	15
1	psoriasis	131	42	4	pharmacokinetics	350	20
1	ankylosing spondylitis	93	51	4	fecal calprotectin	230	24
1	cancer	89	52	4	clinical-response	223	25
1	arthritis	73	56	4	therapeutic drug monitoring	221	26
1	colorectal neoplasia	71	57	4	dose escalation	206	27
1	tuberculosis	69	59	4	serum drug level	187	29
1	severe ulcerative-colitis	65	63	4	trough level	186	30
1	severe crohns-disease	58	68	4	c-reactive protein	166	36
1	psoriatic arthritis	58	69	4	drug monitoring	63	64
1	corticosteroids	58	70	4	loss of response	60	66
1	covid-19	56	74	4	adverse events	52	79
1	active rheumatoid-arthritis	50	80	4	pharmacodynamics	43	88
1	perianal fistula	41	91	5	anti-tnfα	1262	6
1	lymphoma	38	96	5	immunogenicity	173	34
2	infliximab	3305	2	5	biomarker	164	37
2	adalimumab	1077	8	5	cytokine	116	45
2	biologics	725	11	5	immunosuppression	99	49
2	vedolizumab	637	12	5	t-cells	81	54
2	ustekinumab	465	16	5	integrin	58	71
2	azathioprine	453	17	5	gene	58	72
2	tofacitinib	256	23	5	immune response	55	77
2	monoclonal antibody	190	28	5	il-23	40	92
2	janus kinase inhibitor	144	38	5	genome-wide association	37	97
2	certolizumab pegol	142	39	5	nf-kappa-b	36	100
2	immunotherapy	138	40	6	maintenance therapy	1744	5
2	complications	135	41	6	induction therapy	1249	7
2	ct-p13	126	43	6	children	369	19
2	cyclosporine	124	44	6	combination therapy	325	21
2	methotrexate	105	47	6	infection	182	32
2	golimumab	98	50	6	recurrence	170	35
2	etanercept	68	61	6	gut microbiota	111	46
2	natalizumab	56	75	6	rescue therapy	104	48
2	upadacitinib	52	78	6	extraintestinal manifestations	74	55
2	6-mercaptopurine	46	84	6	pregnancy	70	58

2	5-aminosalicylic acid	43	86	6	opportunistic infections	68	62
2	mesalamine	36	98	6	mortality	61	65
3	surgery	298	22	6	switch therapy	57	73
3	endoscopy	182	31	6	apoptosis	56	76
3	colectomy	180	33	6	women	48	82
3	postoperative complications	89	53	6	monotherapy	48	83
3	resection	69	60	6	adults	44	85
3	postoperative recurrence	59	67	6	intestinal inflammation	43	89
3	restorative proctocolectomy	48	81	6	vaccination	42	90
3	pouch-anal anastomosis	43	87	6	susceptibility	40	93
3	anal anastomosis	36	99	6	deep remission	39	94
4	efficacy	936	9	6	adolescents	39	95
